# Edge-subtraction X-ray ptychographic imaging with pink beam synchrotron radiation and a single photon-counting detector

**DOI:** 10.1038/s41598-020-63161-x

**Published:** 2020-04-16

**Authors:** Francesco Brun, Vittorio Di Trapani, Darren Batey, Silvia Cipiccia, Christoph Rau

**Affiliations:** 1 Department of Engineering and Architecture, University of Trieste, Trieste, Italy; 2 National Institute for Nuclear Physics (INFN) - Trieste division, Trieste, Italy; 3 Department of Physical Sciences, Earth and Environment, University of Siena, Siena, Italy; 4 National Institute for Nuclear Physics (INFN) - Pisa division, Pisa, Italy; 5 Diamond Light Source, Harwell Science and Innovation Campus, Fermi Avenue, Didcot, OX11 0QX UK

**Keywords:** Imaging and sensing, Phase-contrast microscopy, Imaging techniques

## Abstract

We present here a new method of performing X-ray edge-subtraction ptychographic imaging by combining multiple harmonics from an undulator synchtrotron source and an energy discriminating photon counting detector. Conventionally, monochromatic far-field X-ray ptychography is used to perform edge subtraction through the use of multiple monochromatic energy scans to obtain spectral information for a variety of applications. Here, we use directly the undulator spectrum from a synchrotron source, selecting two separate harmonics post sample using the Pixirad-1/Pixie-III detector. The result is two monochromatic images, above and below an absorption edge of interest. The proposed method is applied to obtain Au L-edge subtraction imaging of a Au-Ni grid test sample. The Au L-edge subtraction is particularly relevant for the identification of gold nanoparticles for biomedical applications. Switching the energy scan mechanism from a mechanical monochromator to an electronic detector threshold allows for faster spectral data collection with improved stability.

## Introduction

X-ray spectral imaging captures the energy-dependant response of a sample at every imaging element, mapping directly the elemental, chemical or magnetic information to the spatial structure^1,2^. Edge-subtraction imaging is a type of spectral imaging where two images are acquired at two different energies across an absorption edge. Element identification is performed by digital subtraction of the two images, the resultant image is easily segmented and analysed. Although mainly exploited with monochromatic sources, spectral imaging with polychromatic sources is feasible by using energy resolving X-ray detectors^3^.

X-ray ptychography, a scanning coherent small angle scattering technique^4^, is now widely used at synchrotron sources to provide quantitative phase information at the highest resolutions across extended fields of view without the need for high quality optical elements^5^. The method relies on the phasing of diffraction intensities and so it requires a certain degree of spatial and temporal coherence^6,7^. Conventional spectro-ptychography is achieved via an energy scan across an absorption edge and it has successfully been proved both in soft and hard X-ray regime for magnetic domain imaging^8,9^.

Recently, single acquisition K-edge subtraction (KES) ptychography has been demonstrated within a single undulator harmonic using a hyperspectral detector^10^. However, the detector used suffers from a low flux tolerance resulting in relatively long acquisition times. Here we demonstrate a new modality for edge-subtraction ptychography that makes use of multiple undulator harmonics and a multiple threshold X-ray Photon Counting Detector (XPCD) — the Pixirad-1/Pixie-III^11^ — as an harmonic selector. The nature of the Pixie-III is such that the acceptable count rate is significantly higher compared to a hyperspectral detector^12^, resulting in faster acquisition times while still offering energy discriminating capabilities. The detector is capable of operating at more than 500 Hz, with 10^5^ counts s^−1^ pixel^−1^, giving the potential of spectroscopic ptychography at 2.6 · 10^7^ photons mm^−2^s^−1^.

We apply the proposed method to L-edge subtraction (LES) of a Au-Ni test grid as well as to a Siemens star. The experiment was carried out at the I13-1 coherence branchline^13^ of the Diamond Light Source. The undulator spectrum was filtered to isolate two harmonics, above and below the L-edge of Au, and the Pixirad-1/Pixie-III detector was used to separate these harmonics thanks to its tunable energy thresholds. The results from the new method are compared with those obtained with conventional monochromatic LES imaging. We demonstrate firstly that this is possible with the bandwidth of a single undulator harmonic and secondly also that the harmonic selection can be achieved with an electronic threshold configuration, thus removing the need for a monochromator.

## Results

### Threshold settings and detector bandwidth

The Pixirad-1/Pixie-III XPCD coupled to a CdTe sensor was used for the experiment. This detector has two programmable energy thresholds, *T*_*low*_ and *T*_*high*_. The detector can operate in either integration or colour mode. In integration mode a single threshold is applied and a single image of the integration of all the counts above the threshold is produced. In colour mode Pixirad-1/Pixie-III outputs two images in a single acquisition: the first is the integration of the counts between *T*_*low*_ and *T*_*high*_, the second is the integration of the counts above *T*_*high*_. This modality has already been successfully explored to perform KES computed tomography^14^.

**Figure 1:**
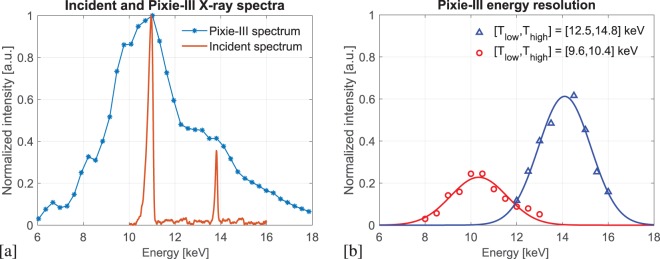
(**a**) Filtered incident pink beam at the sample position as measured with the PCO4000 detector while performing an energy scan (orange, solid) and differential X-ray spectrum as detected by Pixirad-1/Pixie-III (blue, stars) assessed via the threshold scan at a few selected values. (**b**) Detector’s bandwidth when adopting the energy threshold settings to discriminate the 11.0 keV and the 13.8 keV peaks as measured at a few selected energy points.

Discriminating between the two harmonics in the reported experiment requires an accurate setting of the energy thresholds. The undulator X-ray spectrum at the sample, as recorded with an imaging detector (PCO4000 camera) during an energy scan performed using the monochromator, is shown in Fig. 1a. The 4^*th*^ harmonic at 11.0 keV is below the Au L-edge and the 5^*th*^ harmonic at 13.8 keV is above. The bandwidth is 0.33 keV (Full Width Half Maximum - FWHM) for both the 4^*th*^ and 5^*th*^ harmonics, which enforces a spatial resolution limit of 113 nm^6^. Figure 1a also reports the differential X-ray spectrum detected by Pixirad-1/Pixie-III assessed via the so-called threshold scan at a few selected values. The counts above one single energy threshold were recorded for increasing values of this threshold in the range [6.0, 18.0] keV. Then, the difference between two adjacent measured values were used for the reported spectrum.

The optimal levels of *T*_*low*_ and *T*_*high*_ to isolate the harmonics is an optimisation between the finite energy resolution of the detector^15,16^ as reported in Fig. 1b) and the photon statistics. Narrower threshold separations produce images with a higher degree of monochromaticity but at the expense of photon statistics, thus increasing exposure times. Also, having the two thresholds too close can result in unresponsive pixels, because of single pixel miscalibration of the energy thresholds^17^. The following settings were found as optimal: for the “below” image, the detector energy thresholds were set at values of *T*_*low*_ = 9.6 keV and *T*_*high*_ = 10.4 keV, whereas *T*_*low*_ = 12.5 keV and *T*_*high*_ = 14.8 keV were used for the “above” acquisition. It is worth noting that, in order to minimise the contamination between the adjacent harmonics, the energy windows were not centered onto the harmonic peaks but slightly shifted apart, to 10.8 keV and 14.3 keV, respectively.

Figure 1b shows the detector’s bandwidth at the two chosen settings. These were measured by scanning the energy of the X-ray beam with the Si(111) monochromator available at the beamline and dividing the counts between the thresholds by the total number of counts recorded by the detector. The red circles represent the bandwidth with the thresholds set for the harmonic “below” and the blue triangles represent the bandwidth with the thresholds set for the harmonic “above”. For both bandwidths an effective energy spread (FWHM) of 3 keV was measured, which is in agreement with previous similar independent measurements^16^.

### Pink beam LES ptychography of a Au-Ni micro-grid

**Figure 2:**
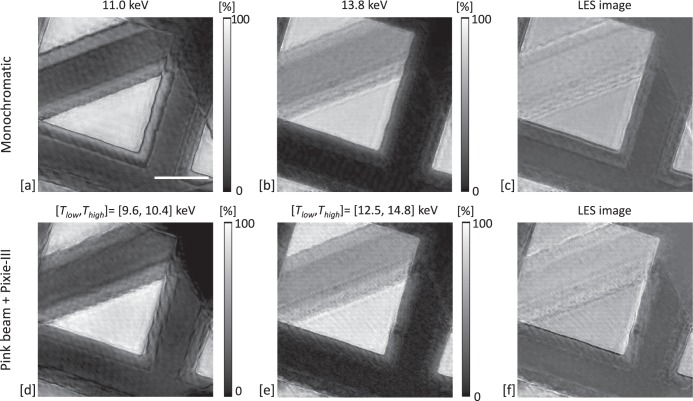
Top row: reference reconstructed images (modulus of the complex-valued ptychographs) acquired with the detector in energy integration mode and by using monochromatic beams at 11.0 keV (**a**) and 13.8 keV (**b**). The digital subtraction of (**a**,**b**) is reported in (**c**). Bottom row: reconstructed images acquired by using pink beam and the detector in colour mode when considering [*T*_*low*_, *T*_*high*_] = [9.6, 10.4] keV (**d**) and [*T*_*low*_, *T*_*high*_] = [12.5, 14.8] keV (**e**). The digital subtraction of (**d**,**e**) is reported in (**f**). [Scale bar = 25.0 m. Color bar for the above and below images representing the X-ray transmission].

The LES imaging of a Au-Ni micro-grid test sample was performed in both monochromatic and pink beam mode. In both cases, two separate acquisitions were required, both above and below the Au L-edge. The illumination was defined by a 5 m diameter pinhole. The acquisitions consisted of a 40 × 40 regular snake scan, with a step size of 2 m (80% beam overlap) and 5 s exposure. For the monochromatic images the Si(111) monochromator was placed in the beam path, the 4^*th*^ (11.0 keV) and the 5^*th*^ harmonics (13.8 keV) were selected in the two consecutive acquisitions. Pixirad-1/Pixie-III was configured in energy integration mode. The top row of Fig. 2 shows the reconstructed object modulus of the complex-valued ptychographs from below and above the absorption edge along with their digital subtraction. For the pink beam acquisition, the monochromator was removed from the beam path. The detector was set in colour mode and the two images were acquired consecutively with the optimised setting for *T*_*low*_ and *T*_*high*_ as described in the previous section. Figure 2d–f shows the reconstructed object modulus for the below, the above, and the corresponding digital subtraction images, respectively. In both monochromatic and pink beam mode the Au is successfully isolated via digital subtraction of the images across the edge.

### Pink beam ptychography of a siemens star

**Figure 3:**
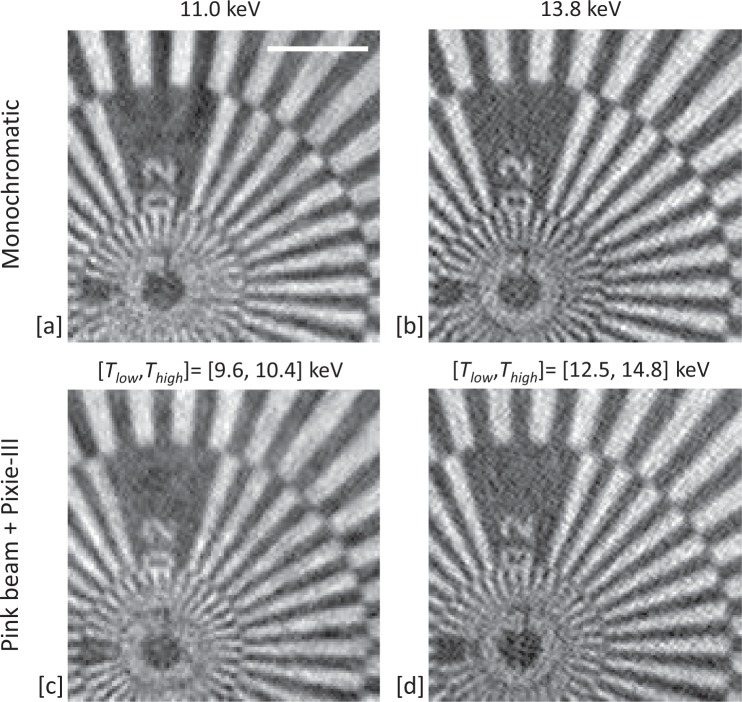
Reconstructed images (phase of the complex-valued ptychographs) of the Siemens star acquired by using monochromatic beams at 11.0 keV (**a**) and 13.8 keV (**b**). Reconstructed images by using pink beam and the detector in colour mode when considering [*T*_*low*_, *T*_*high*_] = [9.6, 10.4] keV (**c**) and [*T*_*low*_, *T*_*high*_] = [12.5, 14.8] keV (**d**). [Scale bar = 2.5 m].

A Siemens star test sample was imaged to evaluate the achievable resolution of the experiment when considering object phase reconstructed images. The acquisition consisted of scanning the sample with the same parameters as for the micro-grid sample, but with 38 × 38 points. Again, for the monochromatic references images the Si(111) monochromator was placed in the beam path and the 4^*th*^ (11.0 keV) and the 5^*th*^ harmonic (13.8 keV) were selected in the two consecutive acquisitions by using Pixirad-1/Pixie-III in energy integration mode. The top row of Fig. 3 shows the reconstructed object phase at the two considered monochromatic energies. For the pink beam acquisition, the monochromator was removed from the beam path and compound refractive lenses (CRLs) were added into the beam path. The detector was set in colour mode and the two images were acquired consecutively with the optimised setting for *T*_*low*_ and *T*_*high*_ as previously described. The reconstructed object phase of the complex-valued ptychographs is shown in Fig. 3. The spatial resolution obtained for the monochromatic and pink beam cases is similar. For each case, the Modulation Transfer Function was formed over a diagonal spoke. The best result of 141 nm was recorded for the reference monochromatic image at 13.8 keV and the worst case of 167 nm was recorded for the pink beam image with detector settings of [*T*_*low*_, *T*_*high*_] = [9.6, 10.4] keV. The achieved resolution of approximately 150 nm is a factor of 67 greater than that resolved by the 5 m probe (beam) size alone. The Universal Quality Index (UQI) was used to estimate the similarity between the two sets of results. The UQI is in the [−1, +1] range and values towards +1 correspond to higher similarities between the images. We obtained for the monochromatic-pink beam comparison a UQI = 0.67 at 11.0 keV and a UQI = 0.70 at the 13.8 keV, which confirm the strong similarity of the results.

## Discussion

We have presented the first results of spectral edge subtraction ptychographic imaging using multiple undulator harmonics and a multi-energy threshold XPCD. LES imaging of a test sample was performed and compared with conventional monochromatic images. The harmonic separation required depends on the specific edge of interest. The considered experiment aims at LES imaging and L-edge is generally weaker than the K-edge so a narrow separation of the harmonics is required. This means that a fine tuning of the detector thresholds is needed. We have proved that, with optimized threshold settings, the quality of the pink beam reconstructed images is comparable to those acquired with monochromatic settings. The edge subtraction process is a precursor for further image segmentation and quantification. Although imperfections in the subtracted images are visible, simple segmentation by gray-level thresholding (slightly above the background noise) accurately isolates the target material.

Pixirad-1/Pixie-III used during the experiment is coupled with a CdTe sensor, which is generally conceived for high-energy imaging^18^. We therefore also expect the methodology outlined here to be valid at higher energies. The use of high-energy X-rays in ptychography is very attractive for their larger penetration power, the depth of focus increases for a given resolution, the dose delivered to the sample is potentially lowered, and shorter wavelengths give access to higher resolution information. Moreover, for the specific case of edge subtraction (ES) ptychography, higher-energy X-rays are required to reach the absorption edges of high-Z elements.

The polychromatic source used in this experiment consists of a comb-like spectrum with energy peaks of different amplitudes. The photon statistics might be significantly different for different energy windows. Filtering of the beam and the detector exposure time can be tuned to mitigate amplitude and consequent signal-to-noise ratio variations. However, in the case of pronounced asymmetry in the energy peaks, attention should be paid to avoid a non-linear response or, even worse, the paralysis of the detector with a high photon flux.

The two images required for the ES were acquired without the need of a monochromator and any mechanical scan of it. This could be beneficial since the stabilization of high-heat-load double-crystal monochromators is one of the most critical challenges for their effective utilization in diffraction-limited storage rings^19^. In addition, having more than two energy thresholds^20^ would allow for the edge subtraction to be performed in a single acquisition. Collecting the data in a single acquisition would be beneficial for the stability and quality of the final results. This is particularly relevant for ptychography, which typically requires longer acquisition times than direct methods. The reduced scanning time of a single-acquisition approach would increase the experimental throughput. Increasing the number of energy thresholds beyond four would allow to target multiple elements at once.

The radiation damage is an important aspect for applications to biomedical imaging. In general ptychography is dose efficient, thanks to the diversity in measurements from the illumination structure and the splitting up of information across the scanning positions. The dose delivered to the sample during the 2D acquisition was estimated from the readout of an ionization chamber placed upstream of the pinhole. A dose of about 200 Gy was calculated for the ptychographic images presented in this article; a value far below the maximum tolerable dose limit described in^21,22^. When considering a 3D acquisition of a hundred projections the delivered dose would be on the order of 2 × 10^5^ Gy, thus still acceptable for the spatial resolution of approximately 150 nm. The methodology described in this work is therefore promising also for biomedical applications of spectro-nanotomography.

## Materials and methods

### Source

The experiment has been developed and conducted at the I13 beamline of Diamond Light Source (DLS). The beamline consists of two independent and complementary branchlines: the coherence branch (I13-1) and the imaging branch (I13-2). The coherence branch is a multimodal, multiscale instrument for reciprocal space imaging: including ptychography^23^, bragg CDI^24^, and bragg-ptychography. The imaging branch is focused on real space imaging. The I13-1 ×-ray source consists of a 2.8 m long undulator with 2.5 cm period. The beamline operates in the 7-24 keV energy range with a distance from the source of 215 m and with a sample-detector distance up to 14.5 m. The Au L-Edge imaging with the proposed technique, requires two isolated harmonics either side of the Au L2 (13.7 keV) and L3 (11.9 keV) edges. During the experiment the undulator was tuned to produce one harmonic above and one harmonic below the Au L-edge with a separation matching the detector energy resolution. The selected harmonics were the 4^*th*^ and the 5^*th*^, respectively at 11.0 and 13.8 keV. This configuration was achieved from a 3 GeV electron storage ring, by opening the undulator gap up to 13.64 mm. In a higher energy storage ring, for a similar insertion device, a smaller undulator gap would have been required to obtain a similar X-ray spectrum. The beam was filtered in order to reduce the contribution from lower and higher harmonics by a combination of metal filters (1.3 mm of pyrolytic graphite plus 0.7 mm of Al) and a Si mirror whose reflectivity drops quickly above 13 keV. The spectrum of the adapted incident beam at the sample is shown in Fig. 1a where two main peaks at 11.0 keV and 13.8 keV, respectively are clearly visible. For the reference images, the X-ray beam was monochromated using a Si(111) double crystal monochromator with 10^−4^ bandwidth.

### Detector

Pixirad-1/Pixie-III is a direct detection XPCD built with a hybrid architecture, where a semiconductor sensor is coupled with flip-chip bonding technique to the ASIC. The sensor is a Schottky type diode array with electron collection on the pixels composed of a thick CdTe crystal (650 m) substrate. A common problem in XPCDs is the limited energy resolution due to charge sharing effects. In Pixie-III this problem is mitigated by summing up the signals of neighbouring pixels, to correctly evaluate the total energy of any event involving up to 4 pixels. This hardware solution improves the energy resolution^11^, and this is beneficial for spectral imaging applications. Pixie-III is a 0.16 mm CMOS ASIC organized as a 512 × 402 matrix of square pixels with 62 m pitch resulting in an active area of 31.7 × 24.9 mm^2^. The readout implements, at single pixel level, two 15-bit counters fed by two independent discriminators with programmable thresholds. During operation the detector is cooled at −30 °C and the CdTe crystal is biased with a working voltage of 400 V. Compared with other XPCDs based on the Medipix ASIC, the Pixie-III is a large area detector (one module of Pixie-III corresponds roughly to a 2 × 2 configuration of Medipix). This avoids the well-known problem of dead gaps in multi-module detectors, due to the fact that up until now it has been possible to make pixel detector read-out chips that can be abutted on three sides only and the fourth side is used for on-chip peripheral logic and wire-bond pads that permit electronic read-out. Dead gaps complicate X-ray imaging because several pixels need to be excluded or inpainted during the reconstruction process^25^. Moreover, miscalibration of the different modules composing a large area detector sometimes occurs and this might affect the quality of the final recorded signal, thus requiring *ad hoc* pre-processing^26^.

### Samples and acquisitions

**Figure 4:**
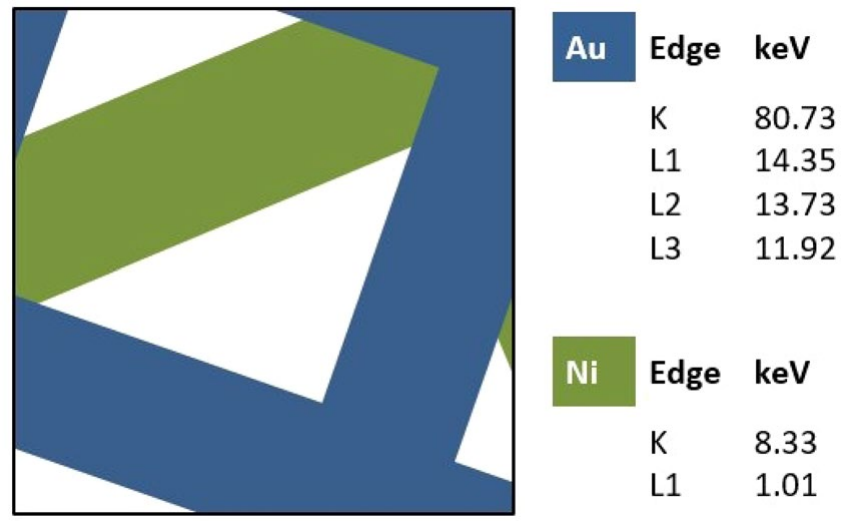
Sketch of the Au and Ni test grid. The values of K- and L-edge for Au and Ni are listed.

A suitable test object composed of a regular microscale grid of Au and Ni component (see schematic in Fig. 4) was scanned to demonstrate the feasibility of Au isolation via L-Edge subtraction. Ni was chosen because the X-ray absorption edges of Ni (K = 8.3 keV and L1 = 1.0 keV) are far away from those of Au. Then a Siemens star was scanned to assess the spatial resolution achievable when considering object phase reconstructed images. The Siemens star is made of Au and has an inner spike width of 50 nm. The detector distance is set by taking into account the resolution required for the lowest energy and the beam size required for the highest energy^7,23^. The optimized experimental configuration resulted in a sample to detector distance of 10 m and a 5 m pencil beam illumination defined by a pinhole.

### Reconstruction and analysis

The ptychographic reconstruction of the acquired patterns was performed using the PtyREX^27^ reconstruction and analysis package available at Diamond Light Source. The software allows for several optimizations^23^ (e.g. correction of motor position misalignment) which compensate for any potential artifacts. Each dataset was processed through 1000 iterations of ePIE^28^ and multi-modal reconstruction with 4 modes was performed^29,30^. For the case of the Siemens star reconstructions, the phase components of the complex-valued ptychographs are shown. The resulting pixel size was 72 × 72 nm for the 11.0 keV case (as well as for the pink beam + Pixirad-1/Pixie-III) and 57 × 57 nm for the 13.8 keV case (as well as for the pink beam + Pixirad-1/Pixie-III). The Universal Quality Index (UQI) was used to quantitatively compare the reference monochromatic image with the proposed pink beam image. For the case of the Au-Ni grid sample, after reconstruction the edge subtraction is performed on the modulus components of the complex-valued ptychographs. Since the pixel size of the reconstructed images is energy-dependent, image registration and calibration were necessary before performing the pixel-by-pixel LES. The resultant image is the difference in absorption across the selected edge.
